# Randomised, double-blind study to evaluate the efficacy of rituximab in the treatment of idiopathic membranous nephropathy: A clinical trial protocol

**DOI:** 10.1371/journal.pone.0320070

**Published:** 2025-03-18

**Authors:** Shinobu Shimizu, Akihito Tanaka, Nao Matsuyama, Fumie Kinoshita, Kazuhiro Furuhashi, Shoichi Maruyama, the PRIME Study Group

**Affiliations:** 1 Department of Advanced Medicine, Nagoya University Hospital, Nagoya, Aichi, Japan; 2 Department of Nephrology, Nagoya University Hospital, Nagoya, Aichi, Japan; 3 Department of Nephrology, Nagoya University Graduate School of Medicine, Nagoya, Aichi, Japan; Guangdong Nephrotic Drug Engineering Technology Research Center, Institute of Consun Co. for Chinese Medicine in Kidney Diseases, CHINA

## Abstract

In Japan, corticosteroid monotherapy has traditionally been recommended as the first-line therapy for membranous nephropathy with nephrotic syndrome. In contrast, except for Japan, rituximab is recommended as the first-line therapy for membranous nephropathy with nephrotic syndrome. This clinical trial aimed to verify the efficacy and safety of the intravenous administration of rituximab without steroids or immunosuppressants as an induction therapy in Japanese patients with idiopathic membranous nephropathy and nephrotic syndrome. This was a multicentre (15 in Japan), placebo-controlled, randomized, double-blind, parallel-group comparative study. A total of 88 patients diagnosed with idiopathic membranous nephropathy and nephrotic syndrome were randomly allocated to rituximab and placebo groups in a 1:1 ratio; rituximab 1,000 mg or placebo IV infusion was administered every 2 weeks for two doses in a double-blinded manner. The primary endpoint was the percentage of patients achieving less than 1.0 g/g creatinine in urine protein/creatinine ratio in random urine at 26 weeks after the first administration of rituximab or placebo. This study was approved by the institutional review boards and conducted in accordance with the Good Clinical Practice guidelines. This study was registered at ClinicalTrials.gov, NCT05914155 and the Japan Registry of Clinical Trials, jRCT2041230037 on 13 June 2023.

## Introduction

Nephrotic syndrome is characterized by massive urinary protein leakage associated with enhanced protein permeability due to glomerular loop disorders and hypoproteinemia (hypoalbuminemia) associated with the leakage. The main diagnostic criteria for adult nephrotic syndrome in Japan are as follows: 1) urine protein ≥3.5 g per day persists (urine protein/creatinine ratio ≥3.5 g/g creatinine in random urine will be handled similarly), and 2) hypoalbuminemia: serum albumin ≤3.0 g/dL (total serum protein ≤6.0 g/dL can be used as reference). Membranous nephropathy is one of the most frequent causative diseases of nephrotic syndrome developing in middle-aged or older individuals, and nephrotic syndrome due to membranous nephropathy is characterized by a relatively slow progression of urine protein and oedema. Approximately 70% of these patients develop nephrotic syndrome [1], and approximately 40% of them develop refractory nephrotic syndrome. Membranous nephropathy is characterized by the thickening of the glomerular basal membrane as a histopathological finding on renal biopsy. Since approximately 20–30% of the patients are spontaneously remitted in the observation of long-term prognosis, membranous nephropathy is said to be relatively benign in glomerular nephritis. However, cases resulting in nephrotic syndrome might have an unfavorable prognosis. A Japanese study has reported that the kidney survival rates of idiopathic membranous nephropathy with nephrotic syndrome are 90.3%, 81.1%, and 60.5% after 10, 15, and 20 years, respectively [2]. On the other hand, a Finnish study on the prognosis of adult patients with idiopathic membranous nephropathy, with urine protein ≥3.0 g per day, reported that 24% of the patients died or had dialysis introduced at year 10 (patient survival rate 80% and kidney survival rate 64%) [3]. Since a decrease in urine protein was reported to be related to an improvement in prognosis, it is considered important to achieve complete or incomplete remission as early as possible.

The 2021 Clinical Practice Guidelines for the Management of Glomerular Disease (KDIGO Guidelines) consider rituximab or calcineurin inhibitors, including cyclosporine and tacrolimus, as first-line drugs for patients with membranous nephropathy at moderate to high risk corresponding to nephrotic syndrome [4]. According to the Japanese guidelines, oral steroid monotherapy is recommended as one of the first-line therapies [5]; however, the KDIGO guidelines do not recommend a single steroid. The Japanese clinical guidelines permit the start of treatment with any of the following: (1) supportive therapy (non-immunosuppressive therapy including diuretics, angiotensin-converting enzyme inhibitors, angiotensin receptor blockers, and antiplatelet drugs); conservative therapies for lifestyle education (smoking cessation and body weight control), salt restriction, and diet; (2) steroid monotherapy; or (3) a combination therapy with steroids and immunosuppressants in addition to (1) supportive therapy. In Japanese epidemiological research, patients using corticosteroids have a high incidence of infection, which is a critical issue in membranous nephropathy [6].

A randomized open-label control study to compare the efficacy of rituximab and conservative therapy (GEMRITUX study) [7] and a randomized open-label non-inferiority study to compare the efficacy of rituximab and cyclosporine (MENTOR study) [8,9] were performed, and rituximab was found to be effective and safe. However, rituximab is not permitted for use in treatment under the Japanese National Health Insurance. Therefore, a prospective placebo-controlled double-blind parallel group study has been planned to examine the therapeutic effect of rituximab in Japanese patients with idiopathic membranous nephropathy with nephrotic syndrome as an investigator-initiated clinical trial. This clinical trial has been named “The multi-center, randomized, double-blind, placebo-controlled study to evaluate the efficacy and safety of rituximab (genetical recombination) for the treatment for idiopathic membranous nephropathy with nephrotic syndrome” (PRIME trial). We discussed the design of this investigator-initiated clinical trial, aimed at evaluating the efficacy and safety of this therapy, in a consultation meeting with the Pharmaceuticals and Medical Device Agency (PMDA), which is the regulatory authority for new drugs in Japan, and the PMDA accepted our proposal.

## Materials and Methods

### Objective, overall design, and framework

The primary objective of this clinical study was to confirm the efficacy and safety of intravenous rituximab in patients diagnosed with idiopathic membranous nephropathy with nephrotic syndrome. The primary endpoint was the percentage of patients achieving incomplete remission type I (ICR I) with the urine protein/creatinine ratio <  1.0 g/g creatinine. The criteria for treatment effects are shown in Table 1.

**Table 1:** Criteria for treatment effects.

CR	Urine protein/creatinine ratio < 0.3 g/g creatinine
ICR I	0.3 g/g creatinine ≤ Urine protein/creatinine ratio < 1.0 g/g creatinine
ICR II	1.0 g/g creatinine ≤ Urine protein/creatinine ratio < 3.5 g/g creatinine
NR	Urine protein/creatinine ratio ≥ 3.5 g/g creatinine
PR	Decrease in urine protein/creatinine ratio from base line ≥ 50%, and urine protein-creatinine ratio 0.3 to 3.5 g/g creatinine

The treatment effect will be evaluated using random urine samples at each point after the administration of the investigational drugs.

CR: Complete Remission, ICR I: Incomplete Remission Type I, ICR II: Incomplete Remission Type II, NR: No Response, PR: Partial Response

**Fig 1:**
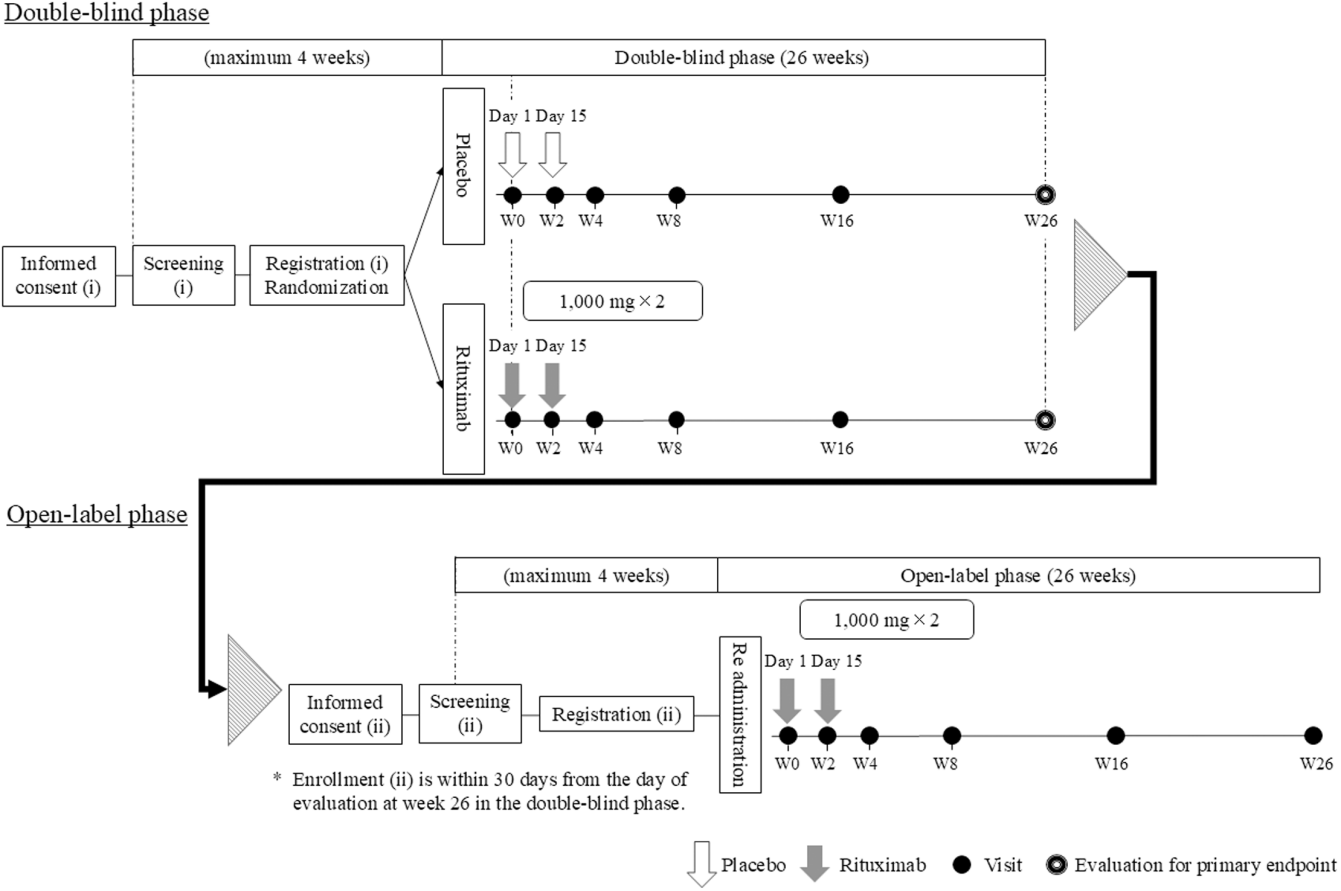
Flowchart of this trial. This shows the schema of this trial, timing of administration of investigational drugs (rituximab or placebo), and assessments and visits for each patient in each phase.

The PRIME study is a placebo-controlled, randomized, double-blind, parallel-group, comparative study, followed by an open-label, single-arm study. In the double-blind phase of this trial, patients will be randomly allocated to rituximab and placebo groups in a 1:1 ratio, and rituximab 1,000 mg or placebo infusion will be administered every 2 weeks for two doses in a double-blinded manner. However, if patients with incomplete remission type II (ICR II) or no response (NR) until the evaluation at week 26 in the double-blind phase wish to move to the open-label phase, and the investigator considers the move necessary, the patient may move to the open-label phase and receive intravenous infusion of rituximab 1,000 mg every 2 weeks for two doses. This move will be performed after the eligibility criteria for re-administration is confirmed. An outline of this clinical trial is shown in Fig 1.

### Eligibility criteria

We will recruit a maximum of 88 patients with nephrotic syndrome due to membranous nephropathy in a double-blind phase. The inclusion and exclusion criteria are follows.

#### Inclusion criteria.

1) Patients who underwent kidney biopsy and are diagnosed as having idiopathic membranous nephropathy prior to obtaining informed consent (i).
2) Patients diagnosed as having nephrotic syndrome prior to obtaining informed consent (i) and who received no steroids or immunosuppressants within 12 weeks prior to obtaining informed consent.
3) Patients with urine protein/creatinine ratio ≥3.5 g/g creatinine at screening (i).
4) Patients with hypoalbuminemia (serum albumin ≤3.0 g/dL) at screening (i).
5) Patients aged 15 years or older at informed consent (i).
6) Patients who provide voluntary written consent after receiving adequate information about this study (legally acceptable representatives should also give consent to patients under 18 years old, and informed assent should be obtained from children).

#### Exclusion criteria.

1) Primary nephrotic syndrome other than membranous nephropathy (IgA nephropathy, minimal change disease, focal segmental glomerulosclerosis, etc.) and secondary nephrotic syndrome (autoimmune disease, metabolic disease, infection, allergic/hypersensitive disease, tumour, and drug-induced disease).
2) Lower renal function (estimated glomerular filtration rate (eGFR) < 30 mL/min/1.73 m^2^ based on the Chronic Kidney Disease Epidemiology Collaboration (CKD-EPI) equations) at screening (i).
3) Patients who have used anti-CD20 antibody, including rituximab, for idiopathic membranous nephropathy.
4) Participation in another clinical study within 12 weeks prior to obtaining informed consent (i) or participation in another study.
5) Patients with a history of renal transplant.
6) Patients with poorly controlled diabetes (HbA1c value of 8.0 or higher).
7) Patients who have or are suspected of having an active infection at the time of informed consent (i).
8) Patients who test positive for HBs antigen, and HBs, HBc, and/or HCV antibodies (patients with positive HBs antibody and/or HBc antibody can be enrolled only when HBV-DNA test is negative by taking appropriate measures, such as periodic monitoring of HBV-DNA and AST/ALT; patients with positive HCV antibody can be enrolled only when HCV-RNA test is negative), or patients with positive HIV or HTLV-1 antibody at the time of screening (i).
9) Patients with leukopenia (less than 2,000/mm^3^), neutropenia (less than 1,000/mm^3^), or lymphopenia (less than 500/mm^3^) at screening (i).
10) Patients with a history of serious hypersensitivity or anaphylactic reaction to one of the ingredients in the investigational drug or murine protein-containing products.
11) Patients who are judged to be having life-threatening nephrotic syndrome by the investigator or sub-investigator.
12) Serious comorbidities (e.g., hepatic, renal (excluding idiopathic membranous nephropathy with nephrotic syndrome), cardiac, lung, haematologic, or brain disease).
13) Female patients who are pregnant, lactating, or potentially pregnant, or patients who are not willing to use contraceptive measures during the study period.
14) Patients who are judged to be unsuitable by the investigator or a sub-investigator

#### Eligibility criteria for re-administration.

The inclusion criteria for open-label phase are as follows.

1) Patients who received the investigational drug in the double-blind phase and showed ICR II or NR until the time of evaluation at week 26, met the following criteria, and wished to have a re-challenge with rituximab.
2) Patients who received no corticosteroids or immunosuppressants between the obtainment of informed consents (i) and (ii).
3) Patients who gave their voluntary written consent after receiving adequate information about the open-label phase of this study (legally acceptable representatives should also give consent to patients under 18 years old, and informed assent should be obtained from children).

#### Exclusion criteria for re-administration.

1) Patients with renal function lowered (eGFR < 30 mL/min/1.73 m^2^ based on CKD-EPI equations) at screening (ii).
2) Participation in another clinical study within 12 weeks prior to providing informed consent (ii) or participation in another study.
3) Patients with a history of renal transplant prior to the obtainment of informed consent (ii).
4) Patients with poorly controlled diabetes (HbA1c value of 8.0 or higher) at the time of screening (ii).
5) Patients who have or are suspected of having an active infection at the time of informed consent (ii).
6) Patients who test positive for HBs antigen, and HBs, HBc, and/or HCV antibodies (patients with positive HBs antibody and/or HBc antibody can be enrolled only when the HBV-DNA test is negative by taking appropriate measures, such as periodic monitoring of HBV-DNA and AST/ALT with reference to the Guidelines for Countermeasures Against the Onset of Hepatitis B; patients with positive HCV antibody can be enrolled only when the HCV-RNA test is negative), or patients with positive HIV or HTLV-1 antibody at the time of screening (ii).
7) Patients with leukopenia (less than 2,000/mm^3^), neutropenia (less than 1,000/mm^3^), or lymphopenia (less than 500/mm^3^) at the time of screening (ii).
8) Patients with an history of serious hypersensitivity or anaphylactic reaction to one of the ingredients in the investigational drug or murine protein-containing products.
9) Patients who are judged to be having life-threatening nephrotic syndrome by the investigator or a sub-investigator at the time of informed consent (ii).
10) Patients with serious comorbidity at the time of informed consent (ii).
11) Female patients who are pregnant, lactating, or potentially pregnant, with obtainment of informed consent (ii), or who are unwilling to use contraceptive measures during the study period.
12) Patients who are judged to be unsuitable by the investigator or a sub-investigator.

### Registration and randomisation

The investigators obtained written informed consent from all eligible patients, who were willing to enroll in the study. After all inclusion and exclusion criteria were confirmed, the investigators registered all items required for enrollment/eligibility confirmation on the electronic data capture system. Patients confirmed to eligible were randomly assigned to the rituximab or placebo group at a ratio of 1:1 in the minimization method according to the following factors: their urine protein/creatinine ratio (<5.0 g/g creatinine/≥5.0 g/g creatinine) at the time of the screening, and use of angiotensin converting enzyme inhibitor or angiotensin receptor blocker at the obtainment of consent (yes/no).

The investigators obtained written informed consent from all eligible patients who showed ICR II or NR until the time of evaluation at week 26 in the double-blind phase, met the re-administration criteria, and wished to have a re-challenge with rituximab. Subsequently, the patients were then registered on the electronic data capture system.

### Intervention and follow-up

Two randomized parallel-group controlled studies were performed on patients with membranous nephropathy with nephrotic syndrome as follows: one of these studies administered 375 mg/m^2^ rituximab intravenous infusion every week for two doses (days 1 and 8) [7], and the other study administered 1,000 mg of rituximab intravenous infusion every 2 weeks for two doses (days 1 and 15) [8,9]. In reference to these clinical studies, the KDIGO guidelines state the dosage and administration of rituximab as 1,000 mg intravenously every 2 weeks for two doses or 375 mg/m^2^ intravenously 1–4 times a week [4]. The dosages and administrations within these ranges may also be effective for Japanese patients with membranous nephropathy and nephrotic syndrome. Therefore, in the double-blind phase, an intravenous infusion of rituximab 1,000 mg or placebo was administered every 2 weeks for two doses. In the open-label phase, an infusion of rituximab (1,000 mg) was administered every 2 weeks for two doses.

All participants were followed-up for 26 weeks in the double-blind and open-label phases after the first dose of the investigational drugs; the planned visits and data collection are presented in Table 2. All data were collected using an electronic data-capture system and checked according to the data management and monitoring plan documented by each manager.

**Table 2:** Protocol for data collection from each patient enrolled in the double-blind and open-label phases of the PRIME study.

Visit	Screening	Start of dosing (Day 1)	Week 2 (Day 15)	Week 4 (Day 29)	Week 8 (Day 57)	Week 16 (Day 113)	Week 26 (Day 183)	At discontinuation
Visit + acceptable range (days)	-28 to -7	0	±3	±3	±7	±14	±14	+14
Dosing of investigational drug		X	X					
Patient background	X							
Serum or plasma pregnancy test	X^a^							
Vital signs^b^	X	X^c^	X^c^	X	X	X	X	X
Oxygen saturation	X	X^c^	X^c^	X				X
12-lead ECG	X							
Laboratory test (blood)^d^	X^e^	X	X	X	X	X	X	X
Laboratory test (random urine)^f^	X	X	X	X	X	X	X	X
Chest X-ray	X						X	X
Blood B cell^g^		X	X	X		X	X	X
Human anti-chimeric antibodies^g^		X				X	X	X
Blood drug level^g^		X^h^	X^h^	X	X	X	X	X
Concomitant drug/therapy	X	X	X	X	X	X	X	X
Adverse events		X	X	X	X	X	X	X

^a^Performed only in women with childbearing potential (women with childbearing potential are those meeting none of the following conditions: 1) had their last menstrual period at least one year ago, 2) underwent hysterectomy, and 3) underwent bilateral oophorectomy).

^b^Systolic and diastolic blood pressure, pulse rate, and body temperature.

^c^Measured within 30 min before starting the study drug administration, immediately before changing the infusion rate, at discontinuation/deceleration of administration, before resuming administration, within 10 min of the end of administration and an hour after the end of administration.

^d^Red blood cell count, white blood cell count, white blood cell differential count (neutrophil, lymphocyte, eosinophil, basophil, monocyte), platelet, haemoglobin, haematocrit, total protein, serum albumin, total bilirubin, direct bilirubin, aspartate amino transferase, alanine amino transferase, gamma-glutamyl transpeptidase, alkaline phosphatase, triglycerides, low-density lipoprotein cholesterol, choline esterase, uric acid, blood urea nitrogen, serum creatinine, creatine kinase, eGFR based on CKD-EPIcr equations, C-reactive protein, blood glucose, and electrolytes (Na, K, Cl, Ca, P, and Mg).

^e^HbA1c and infection tests (HBs antigen, HBs antibodies [HBV-DNA quantification if positive], HBc antibodies [HBV-DNA quantification if positive], HCV antibodies [HCV-RNA quantification if positive], HIV antibodies, and HTLV-1) have been added.

^f^Urine protein, creatinine, glucose, occult blood, and pH

^g^The results will not be disclosed until all the analysis data are fixed.

^h^Blood was collected within 15 min before the start of dosing and 15 min after the end of dosing.

### Primary, secondary, exploratory, and safety endpoints

Regarding the significance of achieving complete remission (CR) or ICR I, a decrease in urine protein level has been reported to be related to an improvement in prognosis [2]. Therefore, it is important to ensure that the patients achieve CR or ICR I as early as possible. In the Japanese guidelines, the evaluation of treatment effects after 1 or 6 months is considered a criterion for deciding the treatment strategy [5]. In contrast, it is known that B cells are depleted for at least 6 months by the administration of 1,000 mg rituximab via intravenous infusion every 2 weeks for two doses (days 1 and 15) for refractory pemphigus vulgaris and pemphigus foliaceus [10]. Therefore, week 26 (month 6) from the start of treatment was set as the point of evaluation to confirm the remission effect of rituximab. Accordingly, the percentage of patients achieving ICR I (criteria shown in Table 1) by week 26 of rituximab administration was defined as the primary endpoint.

The secondary endpoints included: 1) percentage of patients who achieved CR, ICR I, ICR II, NR, or PR (each criterion is shown in Table 1) at each assessment time point; 2) duration before achieving CR, ICR I, ICR II, or PR; 3) urine protein/creatinine ratio; 4) estimated glomerular filtration rate; 5) B cell (CD19-positive and CD20-positives) counts; 6) expression of human anti-chimeric antibodies; and 7) serum rituximab levels. The safety endpoints included adverse events, vital signs, oxygen saturation, and laboratory test results.

### Sample size and statistical analysis

In a cohort study on idiopathic membranous nephropathy by Nagoya University (Nagoya cohort), there were 14 out of 64 (21.9%) patients with membranous nephropathy with equivalent to nephrotic syndrome using no steroids or immunosuppressants (data not disclosed). Therefore, the percentage of patients achieving ICR I 6 months after the initial treatment in the placebo group was expected to be approximately 22% in this study. In addition, as there have been no prior clinical studies on rituximab monotherapy in Japan, the expected efficacy of rituximab is presumed to be 52%. For 80% power with a two-sided significance level of 5%, 40 cases per group were required. Estimating discontinuation/dropout to be approximately 10%, the target number of cases was defined as 88 in the two groups.

We treated this as a safety analysis set in which patients were enrolled for the double-blind phase and received at least one dose of the investigational drug. The primary analysis of the primary and secondary endpoints will be conducted in the full analysis set, with patients in the safety analysis set excluding patients with significant violations of the protocol (not providing consent or significant violation of the trial procedure) and with no data after administration of the investigational drug.

For analysis of the primary endpoint, the percentage of patients achieving ICR I at week 26 and 95% confidence intervals were calculated. The 95% confidence intervals were calculated using the Clopper-Pearson method. A Chi-square test was performed to compare the two groups when analyzing the double-blind phase. For secondary analyses, the percentages of patients achieving ICR-I before weeks 4, 8, and 16 were calculated in a similar manner. The duration before achieving CR, ICR I, ICR II, or PR was estimated using the Kaplan-Meier method. Summary statistics of urine protein/creatinine ratio, eGFR, B cells, and pharmacokinetics parameter were calculated for continuous variables. All statistical analyses were performed using SAS (SAS Institute Inc., Cary, NC, USA) and Phoenix WinNonlin software (Certara, USA). All statistical tests were two-sided, and P-values < 0.05 were considered statistically significant.

### Monitoring and auditing

The monitoring and auditing personnel will include the systematic independence of investigators and confirm the adequacy of the laws, regulations, study protocol, and standard operating procedures, according to the progress of this trial.

### Dissemination

The results of this clinical study will be presented at conferences, submitted to clinical trial registries (ClinicalTrials.gov and Japan Registry of Clinical Trials), and published in peer-reviewed journals.

### Ethics approval and current status on this trial

The study protocol was in accordance with the Declaration of Helsinki, Good Clinical Practice, and the Pharmaceutical Affairs Act of Japan. The trial protocol was approved by the Nagoya University Hospital Institutional Review Board and the remaining 14 institutions (Mie University Hospital, Kasugai Municipal Hospital, University Hospital, Kyoto Prefectural University of Medicine, Kyushu University Hospital, Kurume University Hospital, Anjo Kosei Hospital, Juntendo University Urayasu Hospital, Osaka University Hospital, Konan Kosei Hospital, Kyoto University Hospital, Fujita Health University Hospital, Kanazawa University Hospital, Asahikawa Medical University Hospital,and Hamamatsu University Hospital). In May 2023, the Ministry of Health, Labor, and Welfare approved a clinical trial notification. The specific dates when participant recruitment was started at each of sites were shown in S1 Table. The start date was 1 June 2023, the first patient completed the registration on 24 June 2023 and was administered the investigational drug (rituximab or placebo) on 30 July 2023. The PRIME study has currently enrolled 40 patients and is still recruiting patients from 15 institutes as of 24 September 2024. We are planning to enroll 88 patients as the full analysis set. The limit of enrollment will be till 31 October 2025, and the planned study will end in 31 December 2026.

## Discussion

Membranous nephropathy is characterized by the thickening of the glomerular basal membrane as a histopathological finding on renal biopsy. One of its causes is considered to be the deposition of granular antigen-antibody reaction products below the epithelial cells, which are produced by the binding of specific antibodies against any antigen or the components in the basal membrane themselves. However, the presence of the antibodies responsible for primary membranous nephropathy has been identified. In 2009, autoantibodies against the type-M phospholipase A2 receptor (PLA2R) antigen, mainly consisting of IgG4, were detected in approximately 70% of patients with primary membranous nephropathy, whereas antibodies were not detected in patients with secondary membranous nephropathy or other diseases [11]. In addition, the positivity rate of blood anti-PLA2R antibody was 53% in Japanese patients with primary membranous nephropathy (n = 100) and 0% in patients with secondary membranous nephropathy (n = 31) [12]. However, a case of secondary membranous nephropathy with positive autoantibodies against PLA2R was reported in 2019 [13], requiring careful interpretation. In 2014, the involvement of another candidate with the responsible antigen, thrombospondin type-1 domain-containing 7A (THSD7A), was also revealed, and an autoantibody against THSD7A was reported to be positive in approximately 5% of patients with primary membranous nephropathy. PLA2R and THSD7A are type I membrane proteins with molecular weights of approximately 180 and 250 kDa, respectively. They are expressed on the cell membranes of podocytes in the glomerular basal membrane. When anti-PLA2R or anti-THSD7A antibodies are produced as autoantibodies (IgG) against these proteins, they bind to PLA2R or THSD7A expressed on podocytes to damage the podocytes via the formation of an immune complex and complement activation.

Rituximab is a mouse-human chimeric anti-CD20 monoclonal antibody that exerts cytotoxicity, including complement-dependent and antibody-dependent cell-mediated cytotoxicities, by binding to the CD20 antigen expressed on mature human B cells. Since it has been clarified that autoantibodies against PLA2R or THSD7A are present in many patients with membranous nephropathy, rituximab is expected to suppress the actions of B cells and reduce the production of autoantibodies against PLA2R or THSD7A, the possible causes of disorders, to exert its action for a long period. The efficacy of rituximab on membranous nephropathy was implied in the GEMRITUX [7] and MENTOR studies [8,9]. Accordingly, it was considered likely that rituximab exerts its effects in idiopathic membranous nephropathy with nephrotic syndrome. In Japan, however, rituximab is not approved for the treatment of idiopathic membranous nephropathy with nephrotic syndrome and is not covered by the National Health Insurance; the evidence is insufficient in Japan.

A prospective placebo-controlled double-blind parallel-group study was conducted to examine the therapeutic effects of rituximab in Japanese patients with idiopathic membranous nephropathy and nephrotic syndrome as an investigator-initiated clinical trial. We held a consultation meeting with the regulatory authority, who agreed to the main design of the PRIME study.

This article describes the design of the PRIME trial protocol. The PRIME trial is a randomized controlled trial of rituximab without steroids or other immunosuppressants for membranous nephropathy with nephrotic syndrome. It is anticipated that the results will provide an adequate basis for marketing approval to Japanese patients with membranous nephropathy and nephrotic syndrome.

There are some limitations to this trial. Firstly, the evaluation periods might be short. In the GEMRITUX study, the rituximab group tended to show effectiveness at 6 months after administration of investigational drugs (rituximab or placebo), with a more pronounced reduction in urine protein observed after the 6 months. However, since treatment outcomes between 1 and 6 months are considered to be a criterion for deciding treatment strategies in Japanese guidelines, we could not extend the evaluation period without administering additional treatment to the placebo group, and we decided the timing of the primary endpoint at 26 weeks (6 months). Secondly, the latest guidelines do not require the measurement of autoantibodies for PLA2R or THSD7A, which may be useful for diagnosing membranous nephropathy. Rituximab depletes B cells and may reduce the production of autoantibodies against PLA2R or THSD7A. Therefore, the measurement of autoantibodies in participants of a clinical trial may improve the effectiveness of membranous nephropathy with nephrotic syndrome. Future validation with another clinical trial is recommended.

## Supporting information

S1 Table The specific dates when participant recruitment was started at each of the sites.
(DOCX)
