# Supplementary material for: Randomised, double-blind study to evaluate the efficacy of rituximab in the treatment of idiopathic membranous nephropathy: A clinical trial protocol
Source: PLoS One. 2025 Mar 18;20(3):e0320070. doi: 10.1371/journal.pone.0320070 (PMC11918375; doi:10.1371/journal.pone.0320070)
Supplement: S1 Table — The specific dates when participant recruitment was started at each of the sites. (DOCX) [file pone.0320070.s001.docx]

**Supplemental table 1. The specific dates when participant recruitment was started at each of the sites.**

| Institution | Start date of recruitment |
| --- | --- |
| Nagoya University Hospital | June 5, 2023 |
| Mie University Hospital | June 5, 2023 |
| Kasugai Municipal Hospital | June 5, 2023 |
| University Hospital, Kyoto Prefectural University of Medicine | June 5, 2023 |
| Kyushu University Hospital | June 5, 2023 |
| Kurume University Hospital | June 5, 2023 |
| Anjo Kosei Hospital | June 5, 2023 |
| Juntendo University Urayasu Hospital | June 5, 2023 |
| Osaka University Hospital | August 22, 2023 |
| Konan Kosei Hospital | June 5, 2023 |
| Kyoto University Hospital | June 23, 2023 |
| Fujita Health University Hospital | June 23, 2023 |
| Kanazawa University Hospital | June 23, 2023 |
| Asahikawa Medical University Hospital | June 29, 2023 |
| Hamamatsu University Hospital | July 10, 2023 |
